# Cystitis glandularis forming a tumorous lesion in the urinary bladder: A rare appearance of disease

**DOI:** 10.4103/0970-1591.44268

**Published:** 2008

**Authors:** Kazuyoshi Shigehara, Tohru Miyagi, Takao Nakashima, Masayoshi Shimamura

**Affiliations:** 1 Department of Urology, Kanazawa University Graduate School of Medicine, 13-1, Takaramachi, Kanazawa City, Ishikawa, 920-8640, Japan; 2 Ishikawa Prefectural Central Hospital, Ishikawa, Japan

**Keywords:** Cystitis cystica, cystitis glandularis, proliferative cystitis

## Abstract

We report a rare appearance of cystitis glandularis forming a tumorous lesion with blueberry spots in the urinary bladder. A 49-year-old woman was admitted to our hospital with pollakisuria and recurrent gross hematuria. Urine examination showed no pyuria. Computed tomography (CT) scan showed an extravesical invasive mass and cystoscopy revealed a non-papillary tumor with blueberry spots in the bladder. Transurethral resection (TUR) was performed. Histopathological examination revealed cystitis glandularis with cystitis cystica in the part. Postoperative CT scan and cystoscopy showed reduction of the mass in the bladder without any treatments. She is currently well with no evidence of tumor growth three months after TUR.

## INTRODUCTION

Cystitis glandularis is a benign proliferative disorder of the bladder. Cystitis glandularis is not a rare disease, but cases with the appearance of cystitis glandularis forming a tumorous lesion with blueberry spots suspected to be caused by endometriosis were not reported. We report a rare case of this appearance in the urinary bladder.

## CASE REPORT

**Figure 1:**
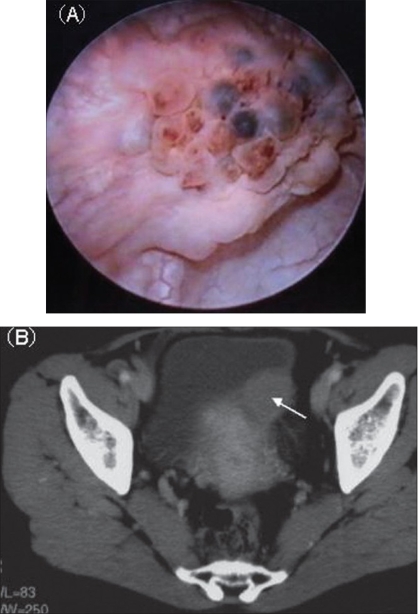
(A) Cystoscopic examination revealed a non-papillary tumor with blueberry spots in the urinary bladder. (B) CT revealed an invasive mass in the bladder

**Figure 2:**
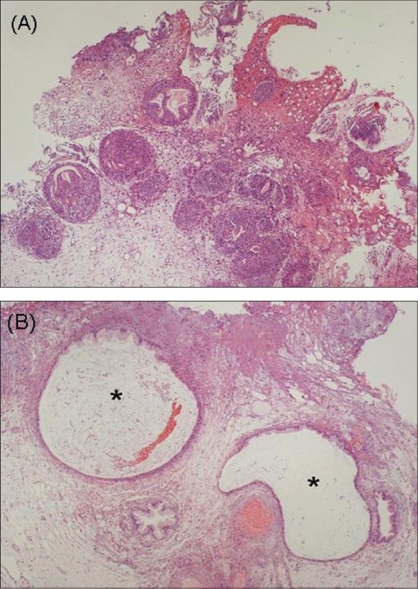
(A) The histopathological examination showed Brunn's nests with glandular metaplasia and cystitis glandularis (H & E, ×4). (B) Cystitis cystica also existed in a part of the specimens (H & E, ×10)

A 49-year-old woman was admitted to our hospital with pollakisuria and recurrent gross hematuria. She had no previous history of recurrent urinary tract infections. She had a past history of right ovariectomy for ovarian cyst 15 years earlier and had received hormonal therapy for endometriosis of the uterus six months prior to presentation. Urine microscopic examination showed slight hematuria (Red Blood Cells: 10^∼^19/hpf) and no pyuria. Blood laboratory data including the tumor markers of carcinoembryonic antigen and carbohydrate antigen 19-9 were within normal limits. Urine cytology revealed no atypical cells and urine culture yielded no microorganisms. Cystoscopy revealed a nonpapillary tumor with blueberry spots on the left posterior wall in the urinary bladder [Figure 1A]. Computed tomography (CT) scan showed an enhanced invasive mass measuring 3 cm in diameter [Figure 1B]. Although the tumor was suspected to be bladder endometriosis, we could not exclude the possibility of a malignant tumor and performed transurethral resection (TUR). Complete resection of the tumor could not be performed because, based on the cystoscopic and radiological findings, it was thought to be an extravesical invasive mass. The histopathological examination showed Brunn's nests with glandular metaplasia and cystitis glandularis, coexistence of cystitis cystica in the part, with invasion of inflammatory cells, but no malignant cells were identified [Figure 2]. Postoperative CT scan showed reduction of the mass in the bladder and only an enhanced thickness of the left wall three months after TUR. Cystoscopy demonstrated only an edematous mucosal change of the resection scar. Urine cytology has showed no atypical cells. She is currently asymptomatic with no evidence of tumor growth and no significant cystoscopic findings at 12 months follow-up.

## DISCUSSION

Cystitis glandularis is not a rare disease, but a case forming a tumorous lesion with blueberry spots suspected to be endometriosis was a rare appearance of the disease not reported in the past literature. Although the etiology of cystitis glandularis remains unknown, a cause has been postulated that chronic stimulation of the bladder mucosa and chronic inflammation due to urinary tract infection may lead to the overproductive changes in the mucosal cells and glandular metaplasia of transitional epithelial cells.[1] Chronic urinary tract infections, inflammation caused by urolithiasis, outflow obstruction and indwelling urethral or suprapubic catheter are risk factors for development of cystitis glandularis. These risk factors were not present in our case. There are no reports of cystitis glandularis being caused by endometriosis of the uterus. But it is reported that secondary inflammatory mass grew in the bladder due to extravesical inflammation such as appendicitis, salpringitis, colon diverticulitis.[2] Chronic inflammation of the bladder by endometriosis of the uterus may result in proliferative change forming an extravesical mass with wall penetration. Moreover, frequent submucosal bleedings occurred by chronic inflammation of the bladder could cause the formation of blueberry spots.

Cystitis glandularis has been regarded as a potentially premalignant condition predisposing to adenocarcinoma of the bladder.[3] In contrast, it was reported that cystitis glandularis was found in 93% of normal autopsied patients.[4] These findings have suggested that cystitis glandularis is a normal variant of bladder epithelium rather than a premalignant lesion. However, these autopsy series had no gross findings and were only microscopic findings. It has been reported that clinically evident disease with macroscopic findings could be a progression of the disease and premalignant lesions.[1]

Only a TUR is performed in most cases for a diagnosis and a treatment of cystitis glandularis. However, there have been some cases in which total cystectomy was performed because of recurrence or continuance of tumor and symptoms despite frequent TUR of the tumor.[5] In our case, although complete surgical removal of the tumor was impossible, The tumor almost disappeared based on the postoperative radiological and cystoscopic findings without any postoperative treatment. So we did not perform bladder biopsy of the resection scar. We think that careful long-term follow-up is necessary in this patient.
